# Objective Rotational Analysis of EVO Toric ICLs Using Infrared Retinal Retroillumination Imaging

**DOI:** 10.3390/jcm14092895

**Published:** 2025-04-23

**Authors:** Antonio Cano-Ortiz, Álvaro Sánchez-Ventosa, Timoteo González-Cruces, Marta Villalva-González, Juan José Prados-Carmona, Rosa Castillo-Eslava, Miguel Ángel Sánchez-Tena, Cristina Alvarez-Peregrina, Alberto Villarrubia-Cuadrado

**Affiliations:** 1 Department of Anterior Segment, Cornea and Refractive Surgery, Hospital La Arruzafa, 14012 Córdoba, Spain; antoniocanoortiz@gmail.com (A.C.-O.); alvarosventosa@hospitalarruzafa.com (Á.S.-V.); timoteogc@gmail.com (T.G.-C.); marta.villalba7@gmail.com (M.V.-G.); rcastillo@hospitalarruzafa.com (R.C.-E.); alvillarrubia@yahoo.com (A.V.-C.); 2 Departamento de Ciencias de la Salud y Biomédicas, Universidad Loyola Andalucía, 41704 Sevila, Spain; 3 Ophthalmology Department, Reina Sofía Hospital, 14004 Córdoba, Spain; 4 Optometry and Vision Department, Faculty of Optics and Optometry, Complutense University of Madrid, 28037 Madrid, Spain; cristina_alvarez@ucm.es; 5 School of Management, Engineering and Aeronautics, Instituto Superior de Educação e Ciências de Lisboa (ISEC Lisboa), 1750-142 Lisboa, Portugal

**Keywords:** ICL, rotational stability, EVO Toric

## Abstract

**Background/Objectives:** To objectively evaluate the rotational stability, refractive predictability, and visual outcomes of toric EVO ICL using automated infrared retinal retroillumination imaging. Setting/Venue: The research was conducted in a specialized ophthalmic surgery center. Design: Longitudinal analytical prospective study. **Methods:** The methodology included preoperative and postoperative assessments of visual acuity, subjective refraction, corneal topography, and anterior segment OCT. The implantation and alignment process utilized advanced digital centration techniques. Postoperative evaluations were conducted at 1 and 3. **Results:** The study found a mean incision surgically induced astigmatism (SIA) of 0.32 D, and a refractive SIA average of 2.02 D, closely matching the preoperative refractive astigmatism (target-induced astigmatism—TIA) mean of 2.07 D, resulting in a correction index (CI) of 0.96. Rotational stability was high, with 72% of lenses showing less than 5° rotation and 96% under 10° at the 1-month follow-up. No significant correlations were observed between lens rotation and postoperative vault size or horizontal compression, indicating independent factors. The discrepancy between theoretical and observed rotations suggested that the calculation method slightly underestimated actual rotation, which did not significantly affect visual outcomes. Graphical analysis demonstrated minimal impact of lens rotation on uncorrected distance visual acuity (UDVA), confirming the procedure’s efficacy and safety. **Conclusions**: Toric EVO ICL implants provide high rotational stability, excellent refractive predictability, and satisfactory visual outcomes. The study underscores the importance of precise implantation and the minimal influence of lens rotation on postoperative refractive errors.

## 1. Introduction

The implantation of phakic intraocular lenses (pIOLs) for the correction of refractive errors is a technique that has gained popularity in recent years due to its efficacy, safety, predictability, and stability [1], as well as its long-term safety [2,3]. Specifically, the correction of astigmatism with EVO ICL lenses has demonstrated high predictability in correcting refractive astigmatism [4,5,6] in both high and low astigmatisms. The proper placement of the lens at the correct meridian is crucial for the correction of refractive cylinders and the stability of the implant itself. It is well-documented that misalignment of the lens position relative to the implantation meridian can affect refractive correction [7].

Several techniques are available to analyze lens centration, such as the one described by Savini in 2019, which involves slit-lamp biomicroscopy photography followed by image processing [8]. However, this method may have potential measurement errors due to the parallax effect when taking the photograph, making it a good approximation method, but perhaps not the most accurate for analyzing the position of the toric lens.

The use of infrared-based retinal retroillumination images has also been described to study the transparency of intraocular media or lenses in the same image [9]. This system may provide a more precise determination of the actual position of the implantation meridian of an intraocular lens. To date, according to our knowledge and literature search, no other study has analyzed the position or stability of a toric phakic intraocular lens implant using automatic analysis of infrared retinal retroillumination images.

Therefore, this clinical analysis aims to evaluate the stability of astigmatism correction with toric EVO ICL pIOLs and their rotational stability over time using infrared-based retinal retroillumination photography.

## 2. Materials and Methods

### 2.1. Preoperative and Postoperative Assessments

All patients underwent preoperative and postoperative assessments, which included the following examinations as part of the routine protocol for refractive surgery patients:

- Visual acuity with and without optical correction.
- Subjective refraction under physiological conditions and cycloplegia.
- Corneal topography using Scheimpflug imaging, determining the simulated keratometric power of the anterior surface (SimK) with a corneal tomographer (Pentacam Oculus, Inc., Arlington, WA, USA).
- Biometry (IOL Master 700, Carl Zeiss Meditech, Jena, Germany).
- Anterior segment optical coherence tomography (AS-OCT) (Casia 2, Tomey Corp., Nagoya, Japan).

Follow-up Assessments

Follow-up assessments included:

- Visual acuity with and without optical correction.
- Subjective refraction under physiological conditions.
- Anterior segment OCT during follow-up (AS-OCT).
- Infrared retroillumination iris imaging with Pentacam AXL Wave during follow-up visits at 1 and 3 months.

### 2.2. Lens Calculation and Implantation

The EVO ICL lens to be implanted was calculated using the commercial online calculator (OCOS, STAAR Surgical, Monrovia, CA, USA), determining the most appropriate lens size and power for each case according white-to-white (WTW) distance and anterior chamber depth (ACD) measured from corneal endothelium to anterior surface of crystalline lens. The pIOL power selected was the one whose residual refractive error was closest to emmetropia, recording the refractive error in spherical equivalent and sphere + cylinder for each subject. The final lens size was chosen based on the OCOS calculator suggestion and the principal investigator’s experience, in addition to the crystalline lens rise (CLR) over the median line between iridocorneal angles, and angle-to-angle distance (ATA) measured with anterior segment OCT (AS-OCT-Casia 2) and corneal tomography based on Scheimpflug imaging (Pentacam AXL Wave).

### 2.3. Surgical Procedure

The surgical procedure used the Calisto digital centration system (Lumera 700, Zeiss). Preoperative data acquisition and surgical planning included keratometry, necessary incision locations for the phakic lens implantation, and the final implantation meridian location according to preoperative theoretical calculations.

After performing the refractive surgery using the standard surgical technique for EVO ICL lens implantation, through a 3 mm temporal incision by the principal surgeon and with the assistance of the Calisto digital centration system (Lumera 700, Zeiss), the same postoperative examination procedure was conducted. The same variables as in the preoperative assessment were measured at 1 and 3 months post-surgery. Data obtained from the research center’s database were entered by the principal investigator’s collaborators into a specially created database, anonymized, and later used for descriptive and statistical analysis.

### 2.4. Lens Orientation Analysis

Infrared retroillumination images were captured using the Pentacam AXL Wave system. Lens orientation was evaluated using automated analysis software integrated in the system, which compares serial images over time. The final orientation of the implanted lens was analyzed using infrared retroillumination photography in medium mydriasis. Medium mydriasis was achieved using a standard pharmacologic protocol (tropicamide 0.5%). The lens orientation was automatically determined by the device software (Pentacam AXL Wave), and the orientation, the difference from the preoperatively planned orientation, and refraction and visual acuity with and without correction were recorded at each visit. This approach established the correction index and refractive predictability of the technique, as well as the impact of lens misalignment or rotation on refraction and visual function of each patient.

### 2.5. Inclusion and Exclusion Criteria

The inclusion criteria were as follows:

- Subjects undergoing refractive surgery with toric EVO ICL phakic lens implantation.
- Preoperative refraction up to −18.00 D of myopia.
- Preoperative corrected distance visual acuity (CDVA) of 1.0 decimal or better.
- Signed informed consent for surgery and data use for future analysis.

Exclusion criteria:

- Unstable refraction in the last 12 months.
- Previous corneal relaxing or perforating incisions.
- Previous ocular surgery.
- Corneal topography showing anterior or posterior curvature abnormalities.
- Ocular or systemic diseases that could affect healing (e.g., connective tissue diseases, diabetes mellitus).

### 2.6. Study Design and Sample Size

This was a longitudinal observational analysis. At least 35 eyes were required for a bilateral variance analysis with a risk α = 0.05 and β = 0.20, a standard deviation of 0.41, and a minimum detectable difference of 0.20, according to sample size calculation using Granmo (Ver. 7.12, Institut Municipal d’Investigació Mèdica, Barcelona, Spain) and data from a preliminary pilot study.

### 2.7. Variables

The variables studied included:

- Pre- and postoperative visual acuity with and without dioptric correction.
- Pre- and postoperative refraction.
- Pre- and postoperative simulated keratometry (SimK).
- Preoperatively calculated and final lens orientation obtained by infrared retroillumination image, and the difference in degrees.
- Effect of lens misalignment on refractive error and uncorrected visual acuity.
- Rotational stability of the implanted lens at 3 months post-surgery.

### 2.8. Statistical Analysis

Statistical analysis of the database included descriptive and correlational statistics between variables, analyzing the residual spherocylindrical refraction based on the misalignment between the planned and achieved orientation post-surgery. This established the refractive correction index, safety and efficacy indices, and the rotational stability of the technique. SPSS Statistics software (IBM, ver. 20.0) was used for statistical analysis, graphical representation, and vectorial analysis of the results.

## 3. Results

A total of 56 eyes from 30 patients were included in this analysis.

### 3.1. Surgically Induced Astigmatism (SIA)

**Figure 1:**
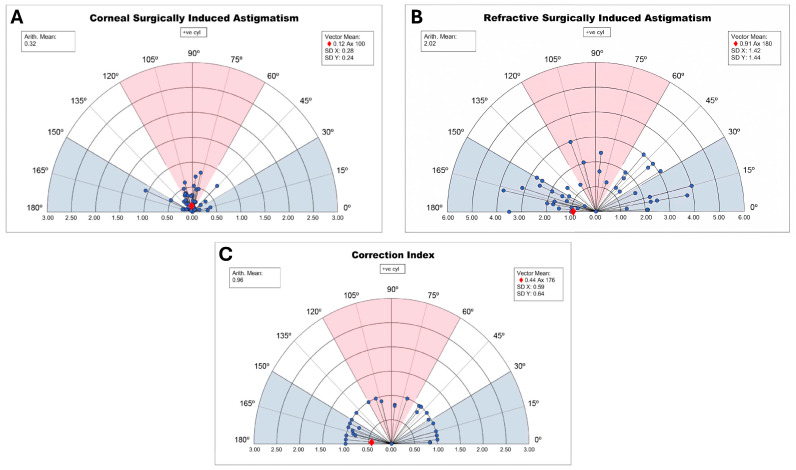
Surgically induced astigmatism and correction index. (**A**): Incisional surgically induced astigmatism. (**B**): Refractive surgically induced astigmatism. (**C**): Correction index (CI). Red = direct astigmatism. Blue = inverse astigmatism; white = oblique astigmatism.

The study found a low surgically induced astigmatism (SIA) with a mean of 0.32 D and a refractive achieved SIA average of 2.02 D, closely matching the mean preoperative refractive astigmatism target of 2.07 D, resulting in a correction index (CI) of 0.96 (Figure 1).

### 3.2. Rotational Stability

**Figure 2:**
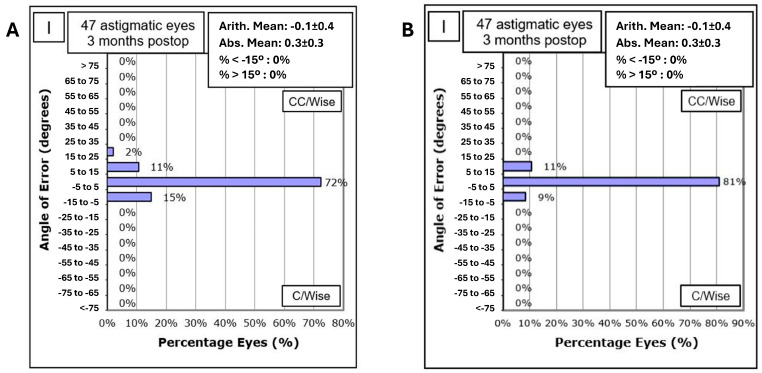
Angle of error. (**A**): Based on retroillumination analysis. (**B**): Based on vector analysis.

Rotational stability was high, with 72% of lenses showing less than 5° rotation and 96% under 10° at the 3 month follow-up according to retroillumination analysis (Figure 2).

The distribution of the angle of error in toric ICL rotation according to vector analysis showed that 81% of eyes had an angle of error within −5° to 5°, 11% between 5° and 15°, and 9% between −15° and −5° at 3 months postoperatively (Figure 2 Angle of error B = according vektor analysis).

### 3.3. Correlation with Vault Size and Horizontal Compression

**Figure 3:**
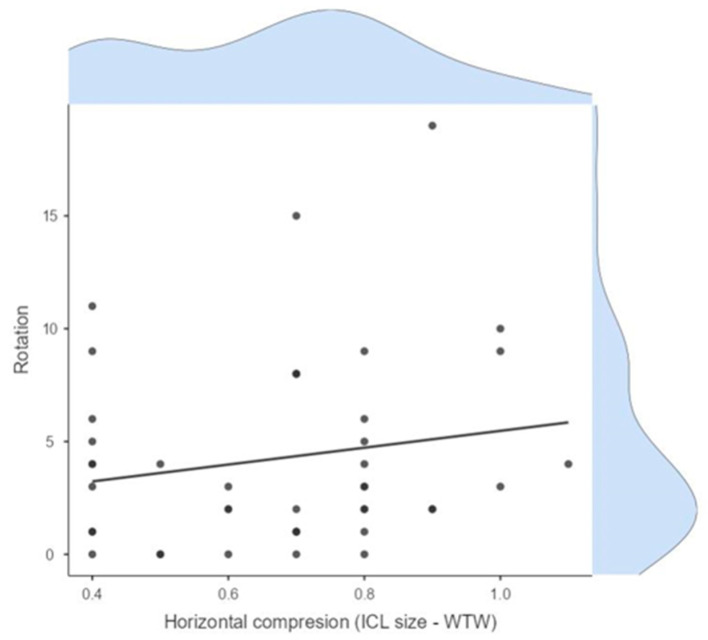
Relationship between horizontal compression (ICL size − WTW) and lens rotation.

No significant correlations were observed between lens rotation and postoperative vault size or horizontal compression, indicating these are independent factors. The linear regression models showed low R-squared values (Figure 3).

### 3.4. Correction Accuracy

**Figure 4:**
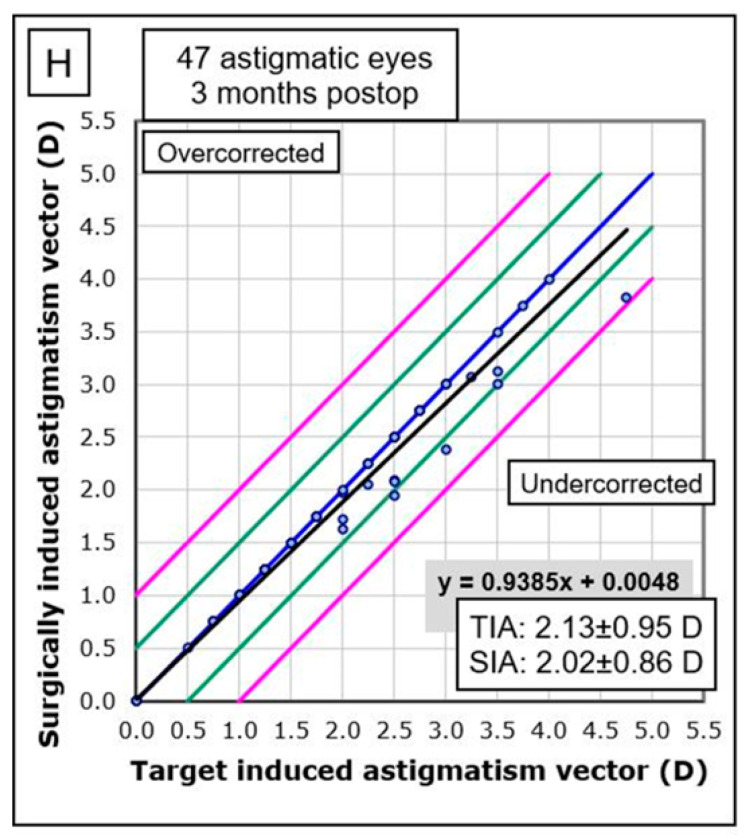
Correlation between target-induced astigmatism (TIA) and surgically induced astigmatism (SIA). Blue line = mean; black line = regression formula line; green line = mean ± 1SD; pink line = mean ± 2SD.

Graphical analysis demonstrated minimal impact of lens rotation on uncorrected visual acuity (UDVA), confirming the procedure’s efficacy and safety. The correlation between the target-induced astigmatism vector (TIA) and the surgically induced astigmatism vector (SIA) was strong, with a regression line close to the line of equality (Figure 4).

### 3.5. Predictability in SEQ and Cylinder

**Figure 5:**
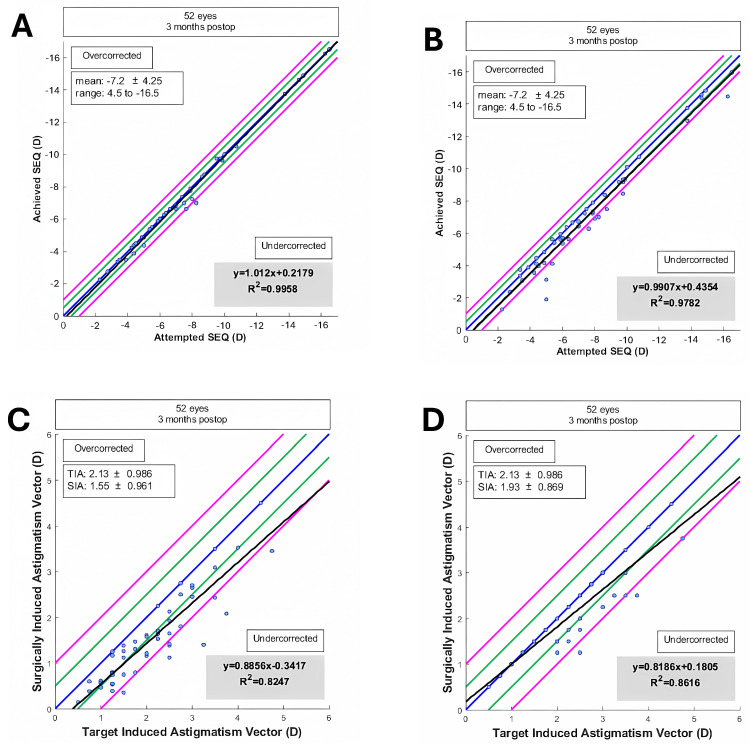
Accuracy of spherical equivalent (SEQ) and cylinder correction. (**A**): SEQ from aberrometric-based refraction. (**B**): SEQ from subjective refraction. (**C**): Cylinder correction from aberrometric-based refraction. (**D**): Cylinder correction from subjective refraction. Blue line = mean; black line = regression formula line; green line = mean ± 1SD; pink line = mean ± 2SD.

The accuracies of the spherical equivalent (SEQ) and cylinder were studied, comparing subjective refraction results and those obtained objectively through aberrometric-based refraction. The regression analysis of achieved versus attempted SEQ showed high predictability with R^2^ values of 0.9782 and 0.9958 for subjective and objective measurements, respectively (Figure 5).

Additionally, the postoperative spherical equivalent refraction distribution indicated that 69% of eyes were within ±0.50 D and 88% within ±1.00 D for subjective refraction. For objective aberrometry-based refraction measurements, 96% of eyes were within ±0.50 D and 98% within ±1.00 D (Figure 5C,D). The accuracy of spherical equivalent (SEQ) was compared between aberrometric-based refraction measurements and between Aberrometric-based refraction (C) and subjective refraction (D).

### 3.6. Summary of Findings

- The corneal SIA generated by the surgeon in EVO ICL toric implantation was low, with a mean of 0.32 D and a vector SIA of 0.12 D at 100°.
- The mean refractive SIA of 2.02 D was similar to the mean preoperative refractive astigmatism of 2.07 D, resulting in a correction index (CI) of 0.96.
- High rotational stability was observed at 1-month post-surgery, with 72% of eyes showing less than 5° rotation and 96% less than 10° rotation.
- A trend of lower UDVA was observed depending on the ICL rotation and the astigmatic power through the parallel coordinates graph.
- Rotational stability of the toric EVO ICL could not be associated with the size of the postoperative vault (Pearson correlation coefficient = 0.10, *p* = 0.50) nor with the horizontal compression of the lens (WTW-ICL size) (Pearson correlation coefficient = 0.17, *p* = 0.25) (Table 1).
- Good concordance was found between the estimated rotation of the toric EVO ICL by vector calculation and that observed by retroillumination. However, the calculated method underestimated the rotation of the pIOL, and it was dependent on cylinder power and exact postoperative refraction, while the observed method by retroillumination was independent of these variables.
- The difference between the TIA and refractive SIA vectors was practically zero, indicating good correction of refractive cylinder.
- High predictability in SEQ and cylinder was achieved, with 69% of eyes within ±0.50 D and 88% within ±1.00 D for subjective refraction, and 96% within ±0.50 D and 98% within ±1.00 D for objective aberrometry measurements.

## 4. Discussion

The results of this study on the rotational stability of the EVO Toric ICL are consistent with findings from previous research, highlighting the efficacy and safety of this technique for astigmatism correction. Although the primary goal was to assess rotational stability, SIA, TIA, and CI were included to provide a comprehensive overview of the refractive outcome profile.

In our study, we achieved high predictability, with 96% of eyes within ±1.00 D and 84% within ±0.50 D of the intended correction. This result is comparable to those reported by Carreras-Díaz et al. [10], who found that 96% of eyes were within ±1.00 D and 84% within ±0.50 D at the last visit. Similarly, Hyun et al. [11] reported that all eyes were within ±1.5 D and 87.5% within ±0.50 D.

The stability in spherical equivalent (SEQ) observed in our study, with values of −0.24 ± 0.49 D at 1 month and −0.23 ± 0.41 D at 12 months postoperatively, aligns with the findings of Zhao et al. (2021) [7], who reported that 100% of eyes were within ±1.00 D and 88.23% within ±0.50 D.

Our results demonstrated a slight undercorrection with a correction index (CI) of 0.96, indicating minimal undercorrection of astigmatism. This finding is similar to that reported by Lee et al. [12], who also found a trend towards undercorrection using vector analysis after TICL implantation.

The significant reduction in manifest astigmatism observed in our study, with approximately 0.50 D of residual astigmatic error, is consistent with the findings of Kamiya et al. [13], who reported that TICL implantation induced corneal astigmatism with a with-the-rule shift of approximately 0.50 D.

Our study showed high rotational stability, with 72% of lenses showing less than 5° rotation and 96% less than 10° at the 1-month follow-up. These results are comparable to those of Shiga et al. [14], who evaluated long-term clinical outcomes after TICL implantation and reported significant rotational stability over an 8–10 year period.

In the study by Wei et al. [15], the prevalence of lenses that required repositioning in the operating room was 0.21% (22 eyes out of 10,258 treated), of which 0.13% (13 eyes) were realigned due to TICL misalignment.

Another significant aspect to highlight is that the rotational stability of T-ICL has been well-demonstrated over the years. However, the objective measurement and monitoring of lens position and potential rotation through our method offers a novel and reliable system with substantial utility for daily clinical practice. Most authors calculate rotation based on the residual refractive error of a patient, but it is crucial to emphasize that postoperatively, there is not always an in-depth search to uncover the minimal residual refractive defect during postoperative refraction. By performing an objective analysis of lens position or utilizing aberrometry-based refraction analysis, we find objective methods to assess the stability of the T-ICL. It is important to recognize that relying solely on residual refraction introduces a bias, as patients with low residual cylinders, around 0.25 to 0.50 D, may not experience any visual function impairment and might be considered emmetropic, when in fact, a slight refractive error persists. Perhaps this emmetropia should be regarded as “functional emmetropia,” but it cannot be used as the sole justification for the stability of a toric lens.

Regarding lens exchange, in Wei et al. [15], 0.09% (9 eyes) of the cases required ICL exchange due to sizing errors. Of these, 0.07% (7 eyes) were due to excessive vault, and 0.02% (2 eyes) were due to TICL misalignment combined with low vault. Packer [1] also reported a low incidence of ICL exchange, with 0.3% (2 eyes out of 629) due to excessive vault after initial repositioning.

The absence of significant postoperative complications in our study supports the findings of Alfonso-Bartolozzi et al. [3], who suggested that the central port design of the V4c ICL prevents cataract development and other complications.

Despite the promising results, this study has several limitations:

- The sample size was relatively small, particularly for eyes with ATR and oblique astigmatism. Larger sample sizes are needed to confirm the findings.
- The follow-up period was limited to three months. Longer follow-up studies are necessary to evaluate the long-term stability and safety of the procedure.
- This study was conducted at a single center, which may limit the generalizability of the results.

## 5. Conclusions

This study contributes significant data on the rotational stability and refractive predictability of the EVO Toric ICL. Our findings indicate:

- High rotational stability, with 72% of lenses showing less than 5° rotation and 96% less than 10° at one month postoperatively.
- Excellent refractive predictability, with 96% of eyes within ±1.00 D and 84% within ±0.50 D of the intended correction.
- A low prevalence of secondary surgical interventions, with only 0.21% of eyes requiring repositioning and 0.09% requiring lens exchange due to sizing errors.

These results affirm the efficacy and safety of the EVO Toric ICL for the correction of astigmatism, supporting its continued use in clinical practice.

## Figures and Tables

**Table 1:** Summary of key results.

Variable	Value/Statistics
Mean Corneal SIA	0.32 D
Refractive SIA	2.02 D
Preoperative Refractive Astigmatism	2.07 D
Correction Index (CI)	0.96
Rotational Stability (<5°)	72%
Rotational Stability (<10°)	96%
Correlation with Vault Size	Pearson r = 0.10, *p* = 0.50
Correlation with Horizontal Compression	Pearson r = 0.17, *p* = 0.25
Predictability in SEQ (Subjective)	R^2^ = 0.9782
Predictability in SEQ (Objective)	R^2^ = 0.9958
SEQ Distribution within ±0.50 D (Subjective)	69%
SEQ Distribution within ±1.00 D (Subjective)	88%
SEQ Distribution within ±0.50 D (Objective)	96%
SEQ Distribution within ±1.00 D (Objective)	98%

## Data Availability

The original contributions presented in this study are included in the article. Further inquiries can be directed to the corresponding author.
